# Pharmacokinetics and Relative Bioavailability of Flavonoids between Two Dosage Forms of Gegen-Qinlian-Tang in Rats

**DOI:** 10.1155/2012/308018

**Published:** 2012-11-01

**Authors:** Chung-Ping Yu, Chi-Sheng Shia, Shang-Yuan Tsai, Yu-Chi Hou

**Affiliations:** ^1^School of Pharmacy, China Medical University, 91 Hsueh-Shih Road, Taichung 40402, Taiwan; ^2^Department of Medical Research, China Medical University Hospital, Taichung 40402, Taiwan

## Abstract

Gegen-Qinlian-Tang (GQT), a popular Chinese medicine prescription, consists of Puerariae Radix, Scutellariae Radix, Coptidis Rhizoma, and Glycyrrhizae Radix. This study investigated the pharmacokinetics of GQT in rats and compared the bioavailability between two dosage forms, that is, traditional decoction (TD) and concentrated powder (CP). Rats were given TD and CP of GQT in a crossover design, and blood samples were withdrawn at predetermined time points. The quantitation methods of ten constituents in two dosage forms of GQT and in serum specimen using HPLC were developed and validated in this study. The pharmacokinetic parameters were calculated using noncompartment model. The results showed that daidzein, baicalein, wogonin, berberine, palmatine, and coptisine were not found in the circulation, whereas the sulfates/glucuronides of daidzein, baicalein, and wogonin were the major forms after oral administration of GQT. Comparison between two dosage forms indicated that the AUC_0–*t*_ of daidzein sulfates/glucuronides after administration of CP was significantly lower than that of TD by 28.9%, whereas the bioavailabilities of baicalin/baicalein and wogonoside/wogonin were comparable between two dosage forms. In conclusion, the major flavonoids of GQT were extensively metabolized into sulfates/glucuronides and present as the major molecules in the circulation. TD of GQT revealed higher bioavailability of daidzin/daidzein than CP.

## 1. Introduction

Gegen-Qinlian-Tang (GQT) was a famous prescription firstly described in *Shang-Han-Lun* (also called “treatise on febrile diseases”) and commonly used to treat virus diarrhea, bacillary dysentery, and general fever clinically [1]. GQT is composed of four Chinese herbs, including Puerariae Radix (roots of *Pueraria lobata* (Willd.) Ohwi, PR), Scutellariae Radix (roots of *Scutellaria baicalensis* Georgi SR), Coptidis Rhizoma (rhizomes of *Coptis chinensis* Franch, CR), and honey-processed Glycyrrhizae Radix (roots of *Glycyrrhiza uralensis* Fisch, GR). Recently, numerous studies have reported beneficial effects of these four herbs and their constituents, such as anti-inflammation [2], neuroprotection [3, 4], and anticancer activities [5–7].

**Figure 1:**
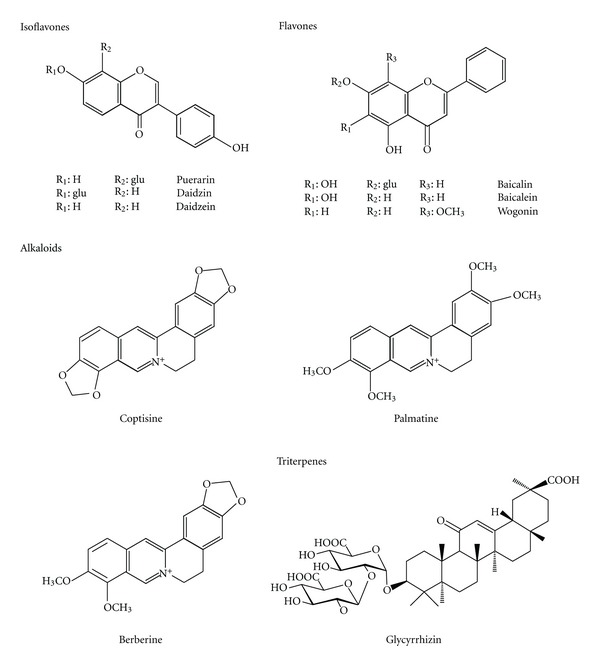
Chemical structures of major constituents in GQT.

The chemical constituents in a Chinese herbal prescription are extraordinarily complex. Understanding the pharmacokinetics of the major constituents in GQT would be helpful to explore the rationale of its clinical implication. The major constituents of GQT include flavonoids such as puerarin, daidzin, daidzein, baicalin, baicalein, and wogonin; alkaloids such as coptisine, palmatine, and berberine; and triterpene glycosides such as glycyrrhizin (chemical structures shown in Figure 1). Based on recent findings of polyphenol pharmacokinetics, it has been generally recognized that most polyphenols are present predominantly as sulfates/glucuronides rather than their parent forms in the bloodstream [8–10]. Besides, the alkaloids, such as berberine, coptisine, and palmatine, the constituents of CR, have been reported to be transformed to sulfates and glucuronides after being absorbed and metabolized by dealkylation [10]. Lately, many endogenous sulfates and glucuronides as well as the conjugated metabolites of xenobiotics have been reported as substrates of multidrug resistance-associated proteins (MRPs) [11, 12] and organic anion transporters (OATs) [12, 13]. We speculate that OATs and MRPs may be associated with the disposition of polyphenol metabolites after oral administration of GQT. Glycyrrhizin, the major constituent of GR, has been found existing as glycyrrhetic acid in the circulation, which was also a putative substrate of OATs and MRPs [14]. Therefore, we hypothesized that the metabolites of GQT might compete with each other for the anion transport mediated by OATs and/or MRPs in intestine, liver, and/or kidney. We thus suspected that the pharmacokinetics and bioavailability of GQT might be different from those of the individual component herbs, for example, PR and SR [8, 9, 14, 15]. 

For a long tradition, Chinese medicines are generally prepared by extracting crude drugs with boiling water and administered as the water extract so called traditional decoction (TD). Nowadays, the dosage form of concentrated powders (CPs) is more popularly prescribed for patients by Chinese medical doctors in Taiwan. Till now, the pharmacokinetics information of both dosage forms of GQT is still lacking, and their bioequivalence remained unknown. Therefore, this study investigated the pharmacokinetics of GQT in rats and, furthermore, the relative bioavailability between TD and CP was evaluated.

## 2. Materials and Methods

### 2.1. Materials and Reagents

The crude drugs, TD and CP of GQT, were supplied by Sun Ten Pharmaceutical Co., Taipei, Taiwan. Briefly, the crude drugs were boiled with water for 4 h and filtered while hot to afford TD. Half volume of TD was then concentrated by spray-drying process with starch as excipient to obtain CP. The origins of SR, PR, GR, and CR were identified by Dr. Yu-Chi Hou and voucher specimens were deposited in China Medical University. Baicalin, baicalein, wogonin, daidzin, and coptisine were supplied by Wako (Osaka, Japan) and their purities were 98%. Berberine (purity 98%) and amyl paraben were obtained from Tokyo Pure Chemical Industries (Tokyo, Japan). Puerarin (purity 80%), daidzein (purity 98%), glycyrrhizin (purity 75%), *β*-glucuronidase (type B-1 from bovine liver), and sulfatase (type H-1 from *Helix pomatia*, containing 14,400 units/g of sulfatase and 574,000 units/g of *β*-glucuronidase) were purchased from Sigma Chemical Co. (St. Louis, MO, USA). Palmatine (purity 97%) was supplied by Aldrich Chemical Co. (Milwaukee, WI, USA). Acetonitrile and methanol (LC grade) were purchased from ECHO Chemical Co. (Miaoli, Taiwan). Ethyl acetate (LC grade) was supplied by Mallinckrodt Baker, Inc. (Phillipsburg, NJ, USA). L(+)-Ascorbic acid was obtained from RdH Laborchemikalien GmbH & Co. KG (Seelze, Germany). Other reagents were HPLC grade or analytical reagent grade. Milli-Q plus water (Millipore, Bedford, MA, USA) was used throughout this study.

### 2.2. Quantitation of the Major Constituents in TD and CP of GQT

The TD (200 *μ*L) containing 1 g/mL of crude drugs was diluted with 200 *μ*L of water and mixed with MeOH (v/v, 3 : 7). After centrifuged, the supernatant (160 *μ*L) was added with 40 *μ*L of amyl paraben solution (100 *μ*g/mL in methanol as internal standard) and 20 *μ*L was subjected to HPLC analysis. The instrumentation included a pump (LC-10ATVP, Shimadzu, Japan), a diode array detector (SPD-M10AVP, Shimadzu, Japan), and an automatic injector (SiL-10AF, Shimadzu, Japan). An Apollo C18 column (4.6 × 250 mm, 5 *μ*m) was equipped with a guard column (4.6 × 50 mm, 5 *μ*m) (Alltech Associates Inc., USA). The mobile phase consisted of acetonitrile (A) and 0.1% phosphoric acid (B). A gradient elution was programmed as follows: A/B: 11/89 (0–10 min), 18/82 (12–25 min), 30/70 (45 min), 43/57 (54 min), 46/54 (59 min), 65/35 (65 min), and 11/89 (70–75 min). The detection wavelength was set at 250 nm and the flow rate was 1.0 mL/min.

For the quantitation of CP, the powders (100 mg) equivalent to 171 mg of crude drugs were accurately weighed and extracted twice with 10 mL of 70% methanol by ultrasonic shaking for 30 min each time and filtered. Sufficient amount of 70% methanol was added to the combined filtrates to make 20 mL. After properly diluted with methanol, the sample was subjected to the HPLC analysis described above.

### 2.3. Animals and Drug Administration

Sprague-Dawley rats were supplied by National Laboratory Animal Center (Taipei, Taiwan) and housed in a 12 h light-dark cycle, constant temperature environment at the Animal Center of China Medical University (Taichung, Taiwan) prior to study. Six male rats weighing 325–490 g were fasted for 12 h before drug administration and food was withheld for another 3 h. Rats were randomly divided into two groups. A single dose of TD and CP of GQT equivalent to 6 g/kg of crude drugs was given to rats via gastric gavage in a crossover design. Water was supplied *ad libitum*. The animal study adhered to “The Guidebook for the Care and Use of Laboratory Animals” published by the Chinese Society for the Laboratory Animal Science, Taiwan. The Institutional Animal Care and Use Committee in China Medical University approved this animal protocol.

### 2.4. Blood Collection

Blood samples (1.0 mL) were collected via cardiac puncture at 15, 30, 60, 120, 360, 720, 1440, 2160, 2880, and 4320 min after dosing and centrifuged at 10,000 g to obtain serum, which was stored at −30°C for later analysis. 

### 2.5. Development and Validation of Quantitaton Method for Serum

The serum specimens were analyzed by HPLC method before and after treatments with *β*-glucuronidase and sulfatase. For the quantitation of glucuronides (G), 200 *μ*L of serum was mixed with 50 *μ*L of *β*-glucuronidase solution (1000 units/mL in pH 5 acetate buffer), 50 *μ*L of ascorbic acid (200 mg/mL) in light protected test tube and incubated at 37°C for 2 h under anaerobic condition, which had been determined by a preliminary study. After hydrolysis, the serum was acidified with 50 *μ*L of 0.1 N HCl and partitioned with 350 *μ*L of ethyl acetate (containing 1 *μ*g/mL of propyl paraben as internal standard). The ethyl acetate layer was evaporated under N_2_ gas to dryness and reconstituted with 50 *μ*L of methanol, then 20 *μ*L was subjected to HPLC analysis. For the quantitation of sulfates/glucuronides, 200 *μ*L of serum was mixed with 50 *μ*L of sulfatase (1000 units/mL, containing 39,861 units/mL of *β*-glucuronidase in pH 5 acetate buffer) and incubated at 37°C for 30 min under anaerobic condition, which had been determined by a preliminary study. The following procedures were the same as that described above for glucuronides. 

An LC-2010C (Shimadzu, Japan) system was used for the analysis and the mobile phase consisted of acetonitrile (A) and 0.1% phosphoric acid (B). A gradient elution was programmed as follows: A/B: 28/72 (0–12 min), 42/58 (28–35 min), 28/72 (40–45 min). The detection wavelength was set at 250 nm and the flow rate was 1 mL/min. 

For the assay of polyphenol free forms, 200 *μ*L of serum sample was subjected to the process described above except the treatments with sulfatase or *β*-glucuronidase. For calibrator preparation, 200 *μ*L of serum was spiked with various concentrations of standards including daidzein, baicalein, and wogonin. The later procedure followed that described above for serum specimen. The calibration graph was plotted by linear regression of the peak area ratios (each standard to internal standard) against concentrations of individual standard.

### 2.6. Validation of the Assay Methods

The system suitability was evaluated through intraday and interday analysis of precision and accuracy. The recoveries of each compound from serum were determined by comparing the peak area of extracted serum standards to those of standards spiked in extracted serum. The LLOQ (lower limit of quantitation) represents the lowest concentration of analyte in a sample that can be determined with acceptable precision and accuracy, whereas LOD (limit of detection) represents the lowest concentration of analysis in a sample that can be detected (with S/N > 3).

### 2.7. Data Analysis

The pharmacokinetic parameters were calculated using noncompartment model with the aid of WinNonlin (version 1.1, SCI software, Statistical Consulting, Inc., Apex, NC). The area under the serum concentration-time curve to the last point (AUC_0–*t*_) was calculated using trapezoidal rule. The peak plasma concentration (*C*_max⁡_) and the time to peak concentration (*T*_max⁡_) were obtained from experimental measurement. Paired Student's *t-*test was used for statistical analysis with significance level set at *P* < 0.05.

## 3. Results

### 3.1. Quantitation of the Major Constituents in TD and CP of GQT

**Figure 2:**
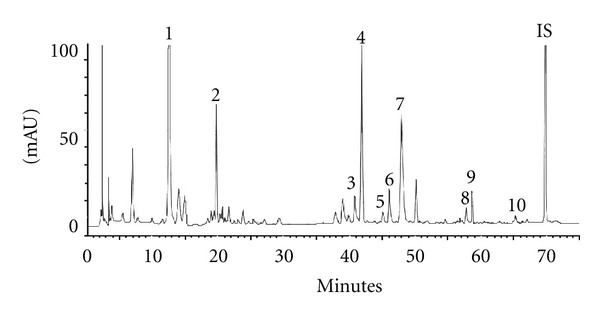
HPLC chromatogram of GQT decoction. 1: puerarin, 2: daidzin, 3: coptisine, 4: baicalin, 5: daidzein, 6: palmatine, 7: berberine, 8: baicalein, 9: glycyrrhizin, 10: wogonin, IS: amyl paraben.

Figure 2 showed the HPLC chromatogram of ten major constituents in GQT decoction, which were satisfactorily resolved within 75 min by a gradient elution.  Table 1 listed the contents of each constituent in TD and CP equivalent to 6 g of GQT. Our quantitation results showed that the contents of ten major constituents in TD and CP were comparable.

### 3.2. Development and Validation of Quantitaton Method for Serum

**Figure 3:**
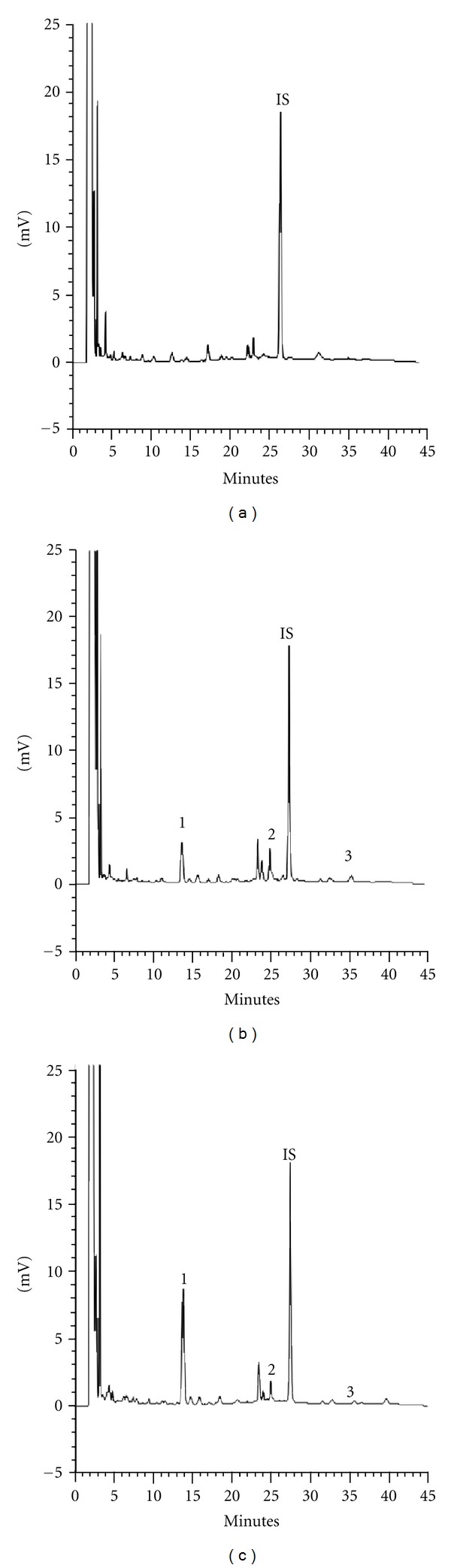
Typical HPLC chromatograms of serum specimens after administration of GQT to rat. (a) Serum specimen, (b) serum specimen after hydrolyzed with glucuronidase, (c) serum specimen after hydrolyzed with sulfatase/glucuronidase. 1: daidzein, 2: baicalein, 3: wogonin and IS: propyl paraben.

The quantitation method for serum specimen was developed and validated in this study. Before enzymatic hydrolysis, the free forms of puerarin, daidzin, daidzein, baicalein, wogonin, coptisine, palmatine, berberine, and glycyrrhizin were not detected. Upon treatment with sulfatase and glucuronidase, daidzein, baicalein, and wogonin emerged. For quantitation of daidzein, baicalein, and wogonin in serum, the calibration curves were in good linearity. The coefficients of variation were less than 9% and the relative errors were below 9% for intraday and interday analysis. The recoveries of daidzein, baicalein, and wogonin from serum were 96.8~100.0%, 83.0~92.3%, and 98.7~106.8%, respectively. The LLOQs were 0.3, 0.2, and 0.1 *μ*g/mL for daidzein, baicalein, and wogonin, respectively, and their LODs were 0.04 *μ*g/mL. Typical HPLC chromatograms of a serum specimen before and after hydrolysis with glucuronidase and sulfatase/glucuronidase are shown in Figure 3.

### 3.3. Pharmacokinetic Parameters of the Conjugated Metabolites of Daidzein, Baicalein, and Wogonin after Administration of GQT in Rats

**Figure 4:**
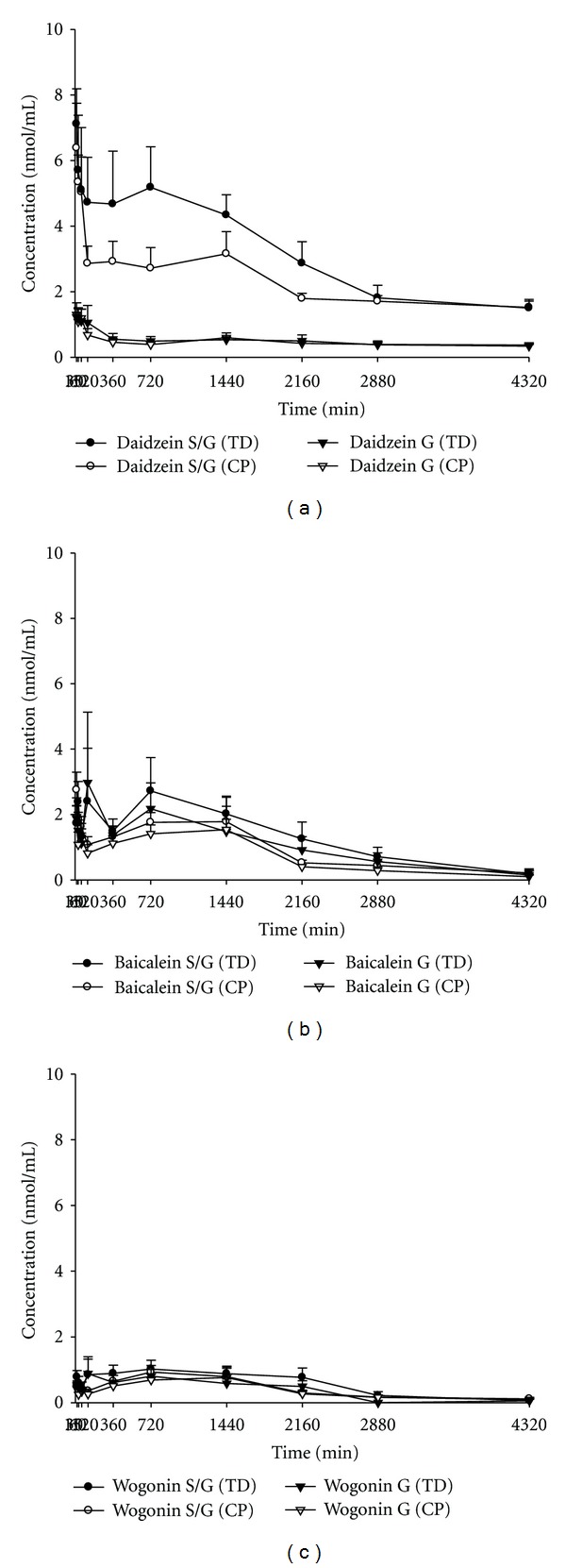
Mean (±S.E.) serum concentration: time profiles of the sulfates/glucuronides (S/G) and glucuronides (G) of daidzein (a), baicalein (b), and wogonin (c) after oral administration of traditional decoction (TD) and concentrated powder (CP) of GQT in six rats.

Figure 4 depicts the serum concentration-time profiles of sulfates/glucuronides (S/G) and glucuronides (G) of daidzein, baicalein, and wogonin in rats after oral administration of TD and CP of GQT. The pharmacokinetic parameters are listed in Table 2. The *C*_max⁡_ and AUC_0–*t*_ of the S/G of daidzein, baicalein, and wogonin were ranked in the order of daidzein S/G > baicalein S/G > wogonin S/G for both dosage forms. The AUC_0–*t*_ of daidzein S/G after administration of CP was significantly lower than that of TD by 28.9%, whereas the AUC_0–*t*_ of baicalein S/G and wogonin S/G after administration of CP and TD were comparable. The relative bioavailabilities of flavonoids between administrations of GQT and PR or SR in rats are shown in Table 3. The AUC_0–*t*_/dose of daidzin/daidzein and baicalin/baicalein in GQT was comparable with those in PR and SR, respectively.

## 4. Discussions

This study is the first work investigating the pharmacokinetics of GQT in rats. The analytical method of serum specimens after administration of GQT was established in this study. The validity of the method was shown by acceptable precision, accuracy, and satisfactory recoveries.

When GQT decoction was orally administered to rats, none of the parent forms of puerarin, daidzin, daidzein, baicalein, wogonin, berberine, palmatine, coptisine, and glycyrrhizin have been detected in serum specimens, indicating that these compounds were not absorbed *per se*. This fact suggested that the *in vitro* bioactivities of those constituents in GQT reported in literature might not predict the *in vivo* effects [16, 17]. What appeared in the circulation was the S/G of daidzein, baicalein, and wogonin, which were found emerging rapidly in serum, indicating that these flavonoids were absorbed quickly and extensively metabolized by conjugation reaction during the first pass [18, 19]. These results were consistent with our previous studies unraveling the pharmacokinetics of PR and SR [8, 9]. The absence of puerarin, daidzin, and glycyrrhizin in serum, which were abundant glycosides in nature, can be explained by their poor lipophilicity which limited their passive diffusion across cell membrane of intestine. Of the isoflavone glycosides, the sugar moiety of daidzin, an O-glycoside of daidzein, was easily hydrolyzed in small intestine to form daidzein, which was able to permeate through the cell membrane of enterocytes [20–22]. As to the metabolic fate of puerarin, a C-glycoside of daidzein, our feces studies have shown that it is hardly hydrolyzed by the gut microflora of rats, rabbits, or humans (data not shown). Besides, the absence of berberine, palmatine, and coptisine in serum specimens can be accounted for by the extensive first pass effect, including dealkylation and conjugation reactions [10]. The pharmacokinetics of the metabolites of these alkaloids has not been investigated because of the unavailability of authentic standards. As to glycyrrhizin, it has been found gradually hydrolyzed to form glycyrrhetic acid, an absorbable aglycone by intestinal bacteria [23]. However, glycyrrhetic acid was not detected in the serum, which might be due to the small dose of GR in GQT and low metabolic rate of glycyrrhizin to form glycyrrhetic acid [14].

Owing to the sulfatase used in this study contained considerable amount of glucuronidase, hydrolysis with sulfatase resulted in the release of both sulfates and glucuronides. The AUC_0–*t*_ of daidzein S/G was 6.5 folds of daidzein G, implying that daidzein S was the major conjugated metabolite of daidzein in blood. This discovery is in good agreement with our previous findings in rats after administration of PR decoction [8]. Interestingly, this was contrary to the metabolic fates of daidzin after intake of soy products, which presented mainly daidzein G in the circulation of female rats, pigs, monkeys, and women [24, 25]. We speculate that the matrix difference between PR and soy products might affect the selective kinetics of conjugation reaction for daidzein in rats. The comparable amounts of baicalein and wogonin liberated between treatments with sulfatase/glucuronidase and glucuronidase in all serum specimens indicated that baicalein G and wogonin G were the major conjugated metabolites, which was consistent with our previous findings in the pharmacokinetic study of SR decoction in rats [15].

When the AUC_0–*t*_ of the S/G of three flavonoids were compared, the systemic exposure was ranked as daidzein S/G ≫ baicalein S/G > wogonin S/G. However, when the ratios of AUC_0–*t*_ of the S/G of each flavonoid to the intake doses including aglycone and glycosides were calculated and compared, their order became daidzin/daidzein > wogonoside/wogonin > baicalin/baicalein, implying that daidzin/daidzein had the highest bioavailability, whereas baicalin/baicalein was the least bioavailable. This fact could be in part accounted for by better dissolution of daidzein than baicalein or wogonin in gastrointestine juice based on its shorter retention time in reversed-phase HPLC chromatogram. On other hand, one possible reason for the poor bioavailability of baicalin/baicalein might be attributed to the poor chemical stability of baicalein, which contains a catechol structure and has been found more degradable than daidzein and wogonin in the artificial intestine juice [20]. 

When the bioavailabilities of each flavonoid were compared between two dosage forms, TD and CP, the significantly lower ratio of AUC_0–*t*_ of daidzein S/G to the intake dose of daidzin/daidzein after administration of CP than that of TD indicated that daidzin/daidzein in TD afforded higher bioavailability. As to the relative bioavailability of baicalin/baicalein and wogonoside/wogonin in two dosage forms, CP and TD were bioequivalent. We thus suggest that, for achieving clinical efficacy, the TD dosage form of GQT is a better choice. 

For the past years, there have been a growing number of *in vitro* bioactivity assays reporting on polyphenols which were commercially available or isolated by phytochemists. For instance, several studies on *in vitro* beneficial activities of the free forms of daidzein, baicalein, and wogonin have been reported [5, 26]. However, based on our pharmacokinetic results showing that the sulfates of daidzein and the glucuronides of baicalein and wogonin were the major forms in the blood, it is likely that the conjugated metabolites of flavonoid play more important role for the pharmacological effects in vascular system than their free forms. In literature, the sulfates/glucuronides of daidzein and glucuronides of baicalein have been shown to exhibit promising activities on breast cancer [26, 27] and bladder cancer [28]. We thus speculate that the clinical efficacy of GQT may be attributed to the bioactivities of these conjugated metabolites of flavonoids.

A few previous studies have reported that herb-herb interaction may occur in Chinese medicine prescription, especially in the decocting process and during absorption or disposition *in vivo* [29, 30]. In this study, in order to assess the relative bioavailability of each flavonoid from GQT, PR and SR, the AUC_0–*t*_/dose of each flavonoid was calculated and the results showed no significant differences between GQT and PR or SR. These facts indicated that the bioavailabilities of these flavonoids from GQT, PR and SR were comparable. We can thus infer that no significant pharmacokinetic herb-herb interaction occurred in the prescription formula of GQT.

## 5. Conclusions

In conclusion, sulfates/glucuronides of daidzein, baicalein, and wogonin were the major forms exposed systemically in rats after oral administration of GQT. Comparison of two dosage forms indicated that TD has higher bioavailability of daidzin/daidzein than CP.

## Figures and Tables

**Table 1:** The contents (mg) of various constituents in traditional decoction (TD) and concentrated powder (CP) equivalent to 6 g of GQT.

Herb	Compound	TD	CP
	Puerarin	71.1	55.6
Puerariae Radix	Daidzin	12.5	10.6
	Daidzein	1.3	1.4

	Bailcalin	80.6	80.4
Scutellariae Radix	Baicalein	3.5	3.6
	Wogonin	1.9	2.3

	Berberine	50.4	43.3
Coptidis Rhizoma	Coptisine	22.7	17.6
	Palmatine	14.0	14.3

Glycyrrhizae Radix	Glycyrrhizin	18.1	16.1

**Table 2:** Comparison of pharmacokinetic parameters between traditional decoction (TD) and concentrated powder (CP) of GQT.

Metabolites	Dosage form	*C*_max⁡_ (nmol/mL)	AUC_0–4320_ (nmol·min/mL)	MRT_0–4320_ (min)
Daidzein S/G	TD	8.9 ± 1.2	13669.7 ± 1704.7^a^	1647.4 ± 103.3
	CP	7.0 ± 0.9	9720.4 ± 853.4^b^ (−28.9%)	1842.7 ± 100.8

Baicalein S/G	TD	4.7 ± 1.3	5445.1 ± 1212.9	1513.7 ± 209.8
	CP	3.2 ± 0.5	3949.7 ± 666.2	1451.3 ± 85.2

Wogonin S/G	TD	1.7 ± 0.4	2314.3 ± 495.4	1409.7 ± 163.9
	CP	1.1 ± 0.3	1816.2 ± 319.7	1476.5 ± 100.9

Data expressed as mean ± S.E.

Means in a column without a common superscript difference, *P* < 0.05.

*C*_max⁡_: peak serum level.

AUC_0–4320_: area under the serum concentration-time curve to 4320 min.

MRT: mean residence time.

**Table 3:** Comparison of bioavailabilities of flavonoids from GQT, PR and SR in rats.

Flavonoids	Treatments	AUC_0–*t*_ (mean ± S.E.)	Dose (*μ*mol/kg)	AUC_0–*t*_/dose
Daidzin/daidzein	GQT (*n* = 6)	11.3 ± 1.5	35.2	0.32 ± 0.04
	PR (*n* = 7)	8.9 ± 1.1	26.6	0.33 ± 0.04

Baicalin/baicalein	GQT (*n* = 6)	5.0 ± 1.3	193.0	0.03 ± 0.01
	SR (*n* = 6)	4.2 ± 0.7	250.9	0.02 ± 0.00

AUC_0–*t*_ (*μ*mol·min/mL): area under the serum concentration-time curve of sulfates/glucuronides of daidzein and baicalein from zero to the last point.
